# Kinetics of Neutralizing Antibodies in Patients Naturally Infected by H5N1 Virus

**DOI:** 10.1371/journal.pone.0010864

**Published:** 2010-05-27

**Authors:** Philippe Buchy, Sirenda Vong, Simon Chu, Jean-Michel Garcia, Tran Tinh Hien, Vo Minh Hien, Mey Channa, Do Quang Ha, Nguyen Van Vinh Chau, Cameron Simmons, Jeremy J. Farrar, Malik Peiris, Menno D. de Jong

**Affiliations:** 1 Institut Pasteur in Cambodia, Phnom Penh, Cambodia; 2 HKU-Pasteur Research Centre, Hong Kong SAR, China; 3 Hospital for Tropical Diseases, Ho Chi Minh City, Vietnam; 4 Oxford University Clinical Research Unit, Ho Chi Minh City, Vietnam; 5 Department of Microbiology, University of Hong Kong, Hong Kong SAR, China; 6 Academic Medical Center, Department of Medical Microbiology, University of Amsterdam, Amsterdam, The Netherlands; Institute of Molecular and Cell Biology, Singapore

## Abstract

**Background:**

Little is known about the kinetics of anti-H5 neutralizing antibodies in naturally H5N1-infected patients with severe clinical illness or asymptomatic infection.

**Methods:**

Using H5N1 microneutralisation (MN) and H5-pseudotype particle-based microneutralisation assays (H5pp) we analyzed sera sequentially obtained from 11 severely ill patients diagnosed by RT-PCR (follow-up range 1–139 weeks of disease onset) and 31 asymptomatically infected individuals detected in a sero-epidemiological study after exposure to H5N1 virus (follow-up range: 1–2 month –11 months after exposure).

**Results:**

Of 44 sera from 11 patients with H5N1 disease, 70% tested positive by MN (antibody titre ≥80) after 2 weeks and 100% were positive by 3 weeks after disease onset. The geometric mean MN titers in severely ill patients were 540 at 1–2 months and 173 at 10–12 months and thus were higher than the titers from asymptomatic individuals (149 at 1–2 months, 62.2 at 10–12 months). Fractional polynomial regression analysis demonstrated that in all severely ill patients, positive titers persisted beyond 2 years of disease onset, while 10 of 23 sera collected 10–11 months after exposure in asymptomatically infected individuals tested negative.

**Conclusions:**

Our results indicate that people with asymptomatic H5N1 infection have lower H5N1 antibody titres compared to those with severe illness and that in many asymptomatically infected patients the antibody titer decreased to levels below the threshold of positivity within one year. These data are essential for the design and interpretation of sero-epidemiological studies.

## Introduction

Since 1997, the highly pathogenic avian influenza A (H5N1) virus has spread among poultry and possibly also in wild birds in Asia, Middle-East, Europe and Africa and caused over 470 cases of reported human diseases with more than 280 deaths [1]. The virus evolves as it continues to circulate endemically in poultry in many countries. Continuing occurrences of human infection provides opportunities to H5N1 viruses to adapt to efficient human-to-human transmission. Furthermore, the novel 2009 pandemic H1N1 virus has repeatedly been detected in pigs in many countries including southern China (Peiris – personal communication) and the triple-reassortant gene constellation possessed by this virus has shown a propensity to acquire novel viral haemagglutinin via reassortment [2]. H5N1 virus has occasionally been documented in pigs [3]. Thus, the presence of the pandemic virus in pigs may provide an increased risk of reassortment between avian H5N1 viruses with the pandemic H1N1 virus. This may allow additional opportunities for H5N1 virus adaptation to human-to-human transmission posing potentially new threats to public health. Hence, it is important to conduct sero-epidemiological studies to monitor the extent of asymptomatic or clinically mild H5N1 illness among humans. Such studies will also help define the risk factors for human infections [4]–[10]. Serological methods are essential for the detection of asymptomatic infections and may be helpful to retrospectively confirm suspected cases of H5N1 disease [11]. A significant limitation for the interpretation of serological data, especially for sero-epidemiological studies, is the lack of information on the kinetics of the anti-H5 neutralizing antibody response and particularly that of asymptomatic infections. In this study, we analyzed the characteristics of the antibody response in individuals from Vietnam and Cambodia infected by clade 1 H5N1 virus who experienced a spectrum of illness ranging from fatal or severe disease to moderate illness or asymptomatic infection. Cambodia shares are porous border for humans and poultry with South Vietnam and during the period under study, the H5N1 viruses isolated from southern Vietnam and Cambodia were phylogenetically closely related [12].

## Materials and Methods

### Serum samples

Human sera were collected at the Hospital for Tropical Disease (HTD) Ho Chi Minh City, Vietnam, from patients with severe H5N1 virus infection confirmed by RT-PCR [13]–[15]. Timing of serum collection from hospitalized patients with H5N1 disease (N = 11) between 2003 and 2005 in southern Vietnam are summarised in table 1. Sera from Cambodia (N = 1370) were obtained from people living within 1 km radius of the households of three H5N1 patients. None of the patients were epidemiologically linked [9], [10]. All of them reported having had direct contact with sick/dead poultry a few days to weeks before symptom onset [9], [10], [16]. First blood samples were collected among village participants ∼1–2 months after the date of the patients'deaths. We repeated blood collection for seropositive individuals 9–11 months later. These studies were approved by the Cambodian National Ethics Committee, the Ethics and Scientific Committee of HTD and the Oxford Tropical Research Ethics Committee (OXTREC). A written informed consent form was obtained from all the participants involved in the studies. Since the asymptomatic seropositive individuals were more likely to have independently acquired infection from poultry rather than from the index case, the timing of their infections is imprecise. Since it is not possible to exclude the possibility that these “asymptomatic” individuals may have had a mild influenza-like illness during the period under consideration (or that they acquired the infections months before), we categorise H5N1 seropositives in this group as asymptomatic or mild H5N1 virus infections. Cambodian sera were initially screened using H5 pseudotype particles expressing the haemagglutinin (HA) protein of clade 1 H5N1 virus isolated in Cambodia in 2005 [17]. Positive or indeterminate results were confirmed by haemagglutination inhibition assay (HIA) using horse red blood cells and “standard” microneutralization test (MN). For WHO, an H5N1 infected case is defined by an haemagglutination inhibition (HI) titer (using horse red blood cells) ≥1∶160 and a MN titer ≥1∶80 [11]. However, we considered a seropositive case when the MN titer was ≥1∶160 with an HI test titer <1∶160 but ≥1∶40.

**Table 1:** Characteristics of individuals tested positive by serology in Cambodia and Vietnam.

		Symptomatic cases	Asymptomatic or mild cases
Country of origin		Vietnam	Cambodia
Number of positive H5N1 cases		11	31
deaths		6	0
Median age in years (min - max)		18 (6–35)	12 (2–77)
Gender (male)		33.3%	48.4%
Median follow - up periods (in weeks)		5	28.5
Min - Max in weeks		1–139	7–51
Blood draws (n = 101)		47	54
# of individuals with 1 blood draw (%)		4 (42%)	8 (26%)
# of individuals with 2 or more blood draws (%)		7 (58%)	23 (74%)
# of individuals with 3–12 blood draws (%)		5 (45%)	0

### H5 pseudotyped particle-based neutralization assay

H5 haemagglutinin pseudotyped lentiviral particles (H5pp) were produced, titrated and used as described previously [17]. These H5pp were used in place of H5N1 virus but the other steps of the procedure followed those of the conventional microneutralization (MN) procedure using Madin-Darby canine kidney (MDCK) cells (American Type Culture Collection, Manassas, VA). In contrast to conventional MN tests, the neutralization of infection in the H5pp test was detected by measuring the reduction of end-point chemoluminescent signal compared to controls done in absence of sera (equivalent to 0% neutralization) and in absence of H5pp (equivalent to 100% neutralization), respectively. Briefly, MDCK cells (4000 cells/well) were seeded the day before infection in white 96-well plates (Perkin Elmer) in 50 µL of complete medium. 10^5^ RLU (“relative” luminescent unit) of H5pp (quantity defined after optimization [18]) were incubated with two-fold serial dilution of serum (starting dilution 1∶20, 60 µL/well total) for 2 h at 37°C (5% CO_2_ incubator). Subsequently, 100 µL of fresh complete medium was added to the virus-antibody mix and 140 µL of the virus-antibody mix was transferred back to the cells after the old cell medium was discarded. After 48h incubation at 37°C (5% CO_2_ incubator), 100 µL of Steady-Glo (Promega) luciferase substrate was added directly. Luminescence was read 15 minutes after addition with either Micro-beta (Perkin Elmer) or Glomax (Promega) plate readers. The neutralization titer was defined as the reciprocal of the dilution that matches the positivity criteria (50% neutralization) after fitting with the Hill model [19].

### Microneutralization assay

One hundred tissue culture infectious dose 50 (TCID50) of A/Vietnam/CL26/2004 (for the tests done on Vietnamese sera), A/Cambodia/Q040547/2006 (for the tests performed on Cambodian sera collected in 2006) and A/Cambodia/R0405050/2007 (for Cambodian sera obtained in 2007) were incubated with serial two-fold dilutions (starting from 1∶10) of each serum for one hour at room temperature prior to addition of the virus-antibody mixture onto MDCK cells. The viruses were chosen for their close genetic and antigenic relatedness to the strains responsible for the infection in patients [20]. Cell monolayers were incubated for further 3–4 days and examined for cytopathic effect. Determination of endpoint neutralizing antibody titers was performed in four wells per dilution. The neutralizing titer was defined as the reciprocal of the highest dilution of serum at which the infectivity of 100 TCID50 of H5N1 virus for MDCK cells was completely neutralized in 50% of the wells. Infectivity was identified by the presence of cytopathic effect on day 4 and the titer was calculated by the Reed-Muench method [21].

### Statistical Analysis

For the purpose of this analysis we pooled serological data obtained by standard MN test from patients with RT-PCR confirmed H5N1 disease in Vietnam as well as asymptomatic infections detected by sero-epidemiological investigations in Cambodia. We used the estimated time interval after known exposure as a surrogate of the symptoms onset for asymptomatic cases. Available measures of antibody titers in each week interval were reported as geometric mean titers (GMTs). Week intervals in which there were no observations were treated as missing values. For H5N1 patients' data, we used fractional polynomial regression as a flexible parametric method for the prediction of the relationship between immune response and time interval. We then plotted the curve along with the confidence interval of the GMTs. Log10 transformed GMTs were calculated for the purpose of plotting the kinetics of the immune responses. For asymptomatic individuals' data, a linear model was fitted to the Cambodia data to assess the significance of the change in titers over weeks. An F statistic at p value <0.05 was considered statistically significant. All statistical analyses, graph construction, and curve-fitting as well as 95% CI were computed using STATA 9.0 (Statacorp., college station,TX, Texas). The Two-sample Wilcoxon rank-sum (Mann-Whitney) test was used to compare small numbers of samples. We assessed correlation between the two assays generating the Spearman's correlation coefficient. We then plotted relative differences in values using the Bland and Altman method [22] to visualize and measure how far apart measurements between the two assays can be.

## Results

A total of 42 individuals including 11 (26%) Vietnamese H5N1-infected patients with severe clinical disease and 31 (72%) seropositive Cambodians with asymptomatic infection or mild disease were analyzed. Of note, 6 of the 31 cases presented with an MN titer ≥1∶160 and an HI titer varying from 1∶40 to 1∶80. Of the 11 Vietnamese patients, 6 died. The Vietnamese H5N1-infected patients with severe disease were older than the Cambodian seropositive asymptomatic individuals (median 18 versus 12 years, p = 0.01). There was no difference in gender proportions between the two groups (Table 1).

The average time of follow-up of 11 Vietnamese patients was 34.2 weeks after symptoms onset (range, 1–139 weeks; median, 5 weeks). Of these 11, seven (64%) were bled twice or more for anti-H5N1 antibody testing at different points in time (median 6 blood draws per patient; range, 2–11) (Tables 2 and 3). Of the 31 seropositive Cambodian individuals who were detected through sero-epidemiological surveys, all but eight were bled twice at 1–2 months (n = 31) and 10–11 months (n = 23) after presumed exposure.

**Table 2:** Time of seroconversion and mean antibodies titers measured 3 weeks after onset of disease or presumed exposure.

		Symptomatic cases		Asymptomatic or mild cases	
		MN*	H5pp*	MN*	H5pp*
**Titers ≥1∶80 among**					
Blood samples in Week 1 of fever onset (n = 6)**		0 (0%)	1 (17%)		
Blood samples in Week 2 of fever onset (n = 10)**		7 (70%)	5 (50%)		
Blood samples in Week 3 of fever onset (n = 5)**		4 (80%)	4 (80%)		
**Titers measured after week 3 of fever onset or presumed exposure**					
Geometric mean titers		372	817	102	137
Min		80	80	80	80
Max		7,943	10,169	2,560	15,375

*MN =  standard microneutralization test; *H5pp =  Pseudotyped H5 particles-based microneutralisation test.

**Only available among H5N1-infected patients.

**Table 3:** Timing of sera collection, neutralizing antibodies titers and clinical outcomes from 11 patients with clinically apparent H5N1 virus infection, 2003/2004.

# sera	Timing of collection (days post onset)	Antibody titers measured by MN* and H5pp*	Outcome	Patient identifier in references	
				[26]	[12]
Cl 1	9	138–5	survived	Patient 5	Patient 6
	12	135–57			
	13	100–208			
	21	501–850			
	31	1259–1055			
	77	1995–2488			
	203	537–1174			
Cl 4	6	1–1	died	Patient 7	n/a
	10	50–7			
	13	32–40			
	17	1–29			
Cl 17	5	45–6	died	Patient 8	n/a
Cl 26	6	1–12	survived	Patient 10	Patient 7
	18	5012–10169			
	28	1413–2323			
	68	537–2465			
	188	316–624			
	420	224–995			
	421	224–219			
	551	224–409			
Cl 36	12	891–753	survived	n/a	Patient 8
	15	7943–5475			
	25	1259–4978			
	33	759–3873			
	45	631–565			
	59	166–473			
	95	398–1892			
	246	251–461			
	400	112–851			
	760	224–282			
	773	72–201			
Cl 100	19	112–863	died	n/a	Patient 3
Cl 101	6	17–54	died	n/a	n/a
Cl 107	7	40–165	died	n/a	Patient 4
	10	158–199			
	11	63–255			
Cl 112	9	122–226	died	n/a	n/a
Cl 114	10	200–34	survived	n/a	n/a
	80	126–527			
Cl 115	5	32–47	survived	n/a	Patient 5
	116	158–133			
	268	126–262			
	416	126–650			
	429	224–821			
	444	158–331			

*MN =  standard microneutralization test; *H5pp =  pseudotyped H5 particles-based microneutralization test.

### Antibody kinetics using classic microneutralisation assay

**Figure 1:**
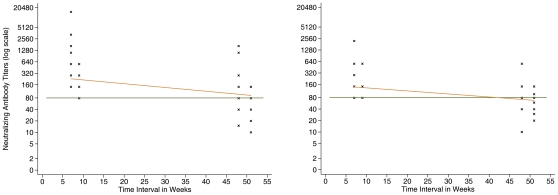
H5 neutralizing antibody titers by H5pp (1a) and MN (1b) tests in 31 asymptomatic/mild individuals. Orange lines: Fig. 1a: Linear regression line (slope of –0.06, p value  = 0.172). Fig1b: Linear regression line (slope of –0.31, p value  = 0.024). Cross points: Seropositive cases' neutralizing antibody titers at Weeks 7, 9, 48 and 51 after exposure (n = 54). Green line: Threshold titer at 80.

In individuals who were asymptomatic or had mild disease, sera collected 10–11 months after exposure showed a 4- to 32- fold reduction in H5N1 neutralizing antibody titers. At this later time point, 10 of 24 individuals (42%) had H5N1 neutralizing antibody titers lower than 1∶80 (ranging from 1∶20 to 1∶40). These ten individuals also had the lowest initial H5N1 neutralizing antibody titers (1∶80) (figure 1). Of the six severely ill patients whose blood was collected during 1–7 days of fever onset (week 1), all tested negative (GMT  = 1∶13.2). Of the 10 blood samples collected among six patients during week 2, seven (70%; 95% CI 34.8–93.3%) tested positive with GMT at 1∶215. Finally, of the five patients tested within week 3, four had antibody titers ≥1∶80 (GMT  = 1∶867). Of the four severely ill patients who were bled for serological testing beyond one year of disease onset (range 1–2.6 years), all tested positive with antibody titers of >1∶80. Of these four, the maximum values of their antibody titers were observed between weeks 2 and 12 of symptom onset (range 1∶1,995–1∶7,943).

### Antibody kinetics using H5 pseudotyped particle-based neutralization assay

**Figure 2:**
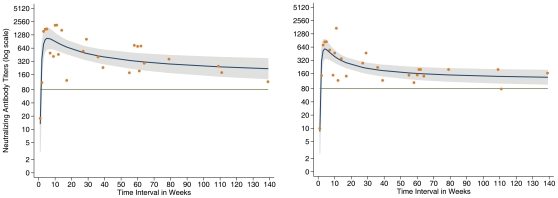
H5 neutralizing antibody titers by H5pp (2a) and MN (2b) tests in 11 severely ill H5N1 patients. Orange dots: Geometric means of titers by week (n = 47). Blue lines: Fractional polynomial regression line. Grey zones: 95% confidence interval around fractional polynomial regression line. Green line: Threshold titer at 80.

**Figure 3:**
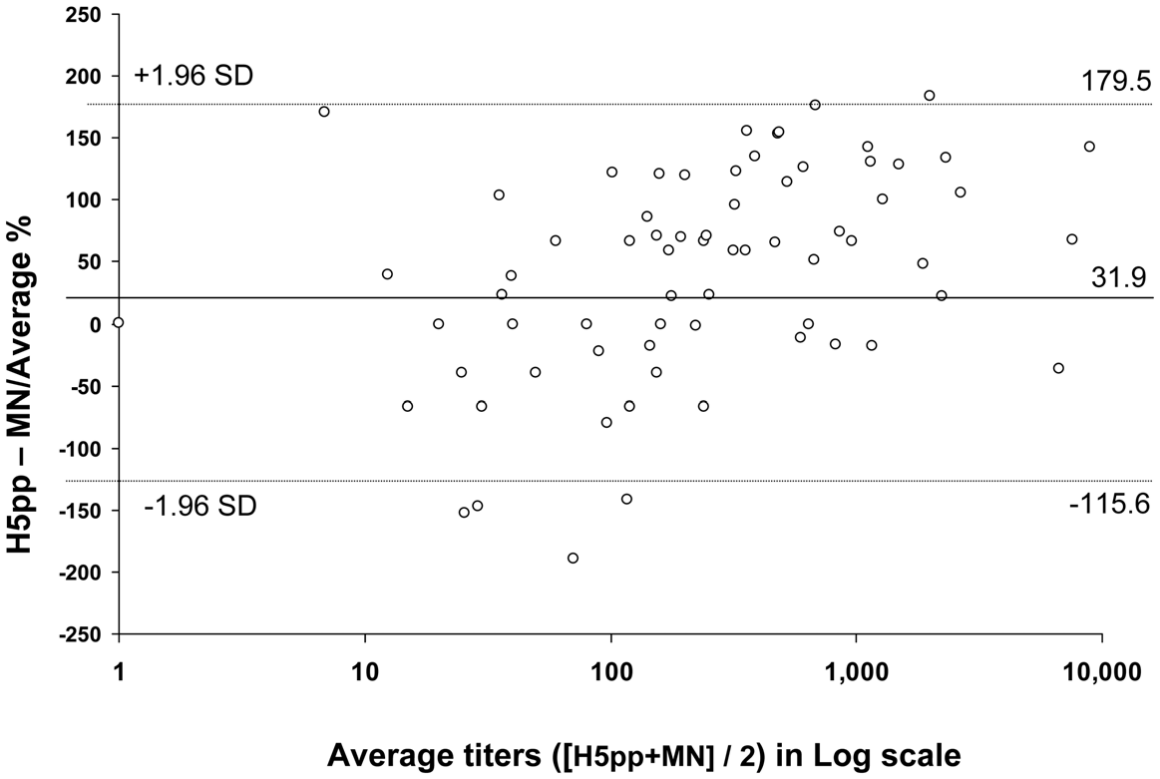
Comparison of H5pp and MN tests by Bland & Altman method. On the x axis, the means of the H5 titers observed with the two methods are shown for individual samples. On the y axis, the difference between the methods divided by the means of the titers presented in percent. The limits of agreements are depicted. A total of 101 sera were included in the analysis. Bland and Altman plot, N  = 101. Bias: 31.9% [95% Confidence Interval  = +17.1% to +46.7%]. Limits of agreement  = −115.6% and +179.5%.

Antibody kinetics measured by H5pp were similar to that from MN assay (Figures 1 & 2). There was good correlation between titers measured by the two methods (Spearman's correlation coefficient of 0.79, p<0.001). Moreover the Bland Altman plot showed that on average H5pp titers were higher by 31.9% (95% CI 17.1%–46.7%) with 95% limits of agreement between −115.6% and +179.5% (units for limits of agreement were expressed in relative differences  =  H5pp titers – MN titers/(H5pp titers +MN titers/2)) (Figure 3). For instance a +179.5% relative difference between the two assays means that H5pp titers were at ∼2 fold-dilution higher than that of MN.

### Regression model analysis

The fractional polynomial regression model for symptomatic individuals predicted a rise of neutralizing antibody titers ≥1∶80 at week 2 and a peak at week 5 or 6 for the 2 assays (Figure 2). Interestingly, the model also predicted positive serology among symptomatic cases using both assays beyond 1 or 2 years after symptoms onset or exposure to H5N1 virus. GMT from sera collected in symptomatic cases (n = 4) at 1–2 months after the symptoms onset tend to be higher than that of asymptomatic cases (n = 31) after 1–2 months of exposure (GMT 540 versus 149; p = 0.084 by MN and GMT 1,266 vs. 228; p = 0.017 by H5pp). In addition, these differences in GMT significantly increased between symptomatic cases (4 patients whose sera were collected at 12–14 months of symptoms onset) and 23 asymptomatic individuals bled at 10–11 months after exposure (GMT 173.0 versus 62.2, p = 0.004 by MN and GMT 420 versus 71, p = 0.010 by H5pp).

## Discussion

This study analysed the kinetics of the anti-H5N1 virus neutralizing antibody response in naturally infected patients with symptomatic and asymptomatic or mild infection. Overall, the titers of antibodies measured by both MN and H5pp assays were generally higher in hospitalized patients with severe H5N1 disease than in subclinically infected individuals. Serological evidence of infection (titer ≥1∶80) was still detectable by both assays in severe H5N1 cases for periods up to the limit of follow-up at 2 years, contrasting with a shorter longevity of detectable antibodies in subclinical cases of whom a substantial proportion declined to titers below 1∶80 within 10 months after presumed exposure. Higher initial titers during the acute phase, rather than differences in decay rate, could explain persistence of significant titers over a longer period of time among sick patients. Indeed, the slopes of the decay of titers were not significantly different between the two groups of cases when using linear regression modelling (data not shown). These findings suggest that conducting serosurveys could still be relevant within several months of the exposure, and that the level of antibody titers in a human population combined with information about the timing of poultry outbreaks and about H5N1 morbidity and mortality among humans could serve as a tool to accurately measure and monitor the extent of transmission as well as the risk factors for transmission and disease in a given area or premise (i.e. markets).

Among patients hospitalized in Vietnam for clinically apparent H5N1 infection, we observed that no neutralizing antibodies were detected during the first week after the onset of the disease while an antibody titer ≥1∶80 was detectable in 70% by day 14 and in 80% of patients by day 21. It cannot be excluded that the patient that did not test positive by day 17 would never seroconvert, as previously described in other patients infected with H5N1- or other influenza viruses [23], [24]. We found that only two-thirds of patients between day 7–14 had serological evidence of infection. This suggests the conduct of serological testing among patients after 2 weeks of disease onset or exposure to the virus, which is consistent with the WHO recommendations.

The regression model used in this study predicts that the antibody titer in clinically ill patients who develop a specific immune response should be higher than 1∶80 by day 14 after onset of symptoms and peaks 2 to 10 weeks after symptom onset. The curve suggests that during the peak, the average expected titer should be between 1∶320 and 1∶640. Interestingly, even 2 years after H5N1 disease, serology should still be positive in most of these severe human cases. To our knowledge, the kinetic data were only documented in 8 severely ill Thai patients naturally infected by H5N1 virus [24]. Our results observed in additional symptomatic patients from Vietnam were similar and consistent with that of Thailand.

For the patients with subclinical H5N1 infection, we observe a titer approximately 3 times lower at the time of recruitment by comparison with patients who developed severe symptoms. The average titer (1∶150) at the time of blood draw (1–2 months after exposure) is just two fold dilution higher than the cut-off value, hence the importance of using neutralization-based methods, which seems to be more sensitive than HI test, and choosing the most appropriate virus for these tests [25]. After approximately 40–45 weeks, the titres in 42% of the subclinical cases had already declined to levels below 1∶80, especially in those who had an initial titer close to the cut-off value. On the basis of the current criteria of serological evidence of H5 infection, about half of subclinically H5N1-infected thus will not be identified if tested one year after exposure. Therefore, seroepidemiological studies should be implemented very early (but not before 3 weeks) after the supposed date of exposure to the H5N1 virus. As a result, the dates of exposure are indispensible for interpreting population-based seroepidemiological data.

The interpretation of our findings is subject to several limitations. The MN tests on the asymptomatic (Cambodian sera) and symptomatic (Vietnamese) sera were done using identical protocols but in two laboratories using different clade 1 H5N1 viruses isolated in Cambodia and Vietnam, respectively. These limitations and the inter-laboratory variations have been highlighted in a recent study [25] but cannot easily be overcome, especially since there are strict restrictions in the movement of clinical specimens and virus isolates across national borders. On the other hand, each laboratory used for its MN tests the viruses autologous to the local infecting virus which would likely increase the sensitivity. Furthermore, these clade 1 viruses in Vietnam and Cambodia were antigenically and phylogenically very closely related to each other. However, to ensure comparability, we retested all sera using the H5pp method at the two laboratories. This assay used the same virus pseudo-particles and the same reagents and was therefore easier to standardize [18]. Given the good agreement and correlation between the standard MN and the H5pp-based microneutralisation test in both sites, we believe our findings accurately describe the overall patterns of the kinetics of H5N1 antibodies. The good correlation of the results between MN and H5pp also provides independent validation for the use of the H5pp assay as a surrogate for MN tests and one that can be done without the need for BSL-3 containment of highly pathogenic viruses. Secondly, although we managed to follow-up many infected individuals, broad variations exist with regard to the number of serum samples collected per person and the period of follow-up. Therefore many of our inferences between time and titer levels, particularly during the early rise of antibody response, were based on small numbers of data-points at each week time interval. It would be important to repeat seroepidemiological surveys with paired samples collected at the early stage of the suspected exposure and two to three weeks later so that the period of infection is more precisely defined.

### Conclusion

There is little data available on the natural history and kinetics of the antibody response to influenza H5N1 infection over time, crucial information required to inform the design of seroepidemiological studies. We have demonstrated a good correlation in the profiles of antibody response of the H5pp and MN titres hence confirming the validity of the H5pp test as a screening test in seroepidemiological studies of H5N1 infection. Our data provide important novel insights into these dynamics of the serological responses in patients with the full spectrum of clinical disease from severe through mild to asymptomatic H5N1 infection. Whilst community based seroepidemiology testing after one year may pick up people with clinically apparent infection it may fail to show the true extent of the community exposure to H5N1 as the antibody response apparently wans faster in those individuals who were mildly symptomatic or asymptomatic. Hence delayed community seroprevalence studies for H5N1 may underestimate the true burden of human infection.
